# PHOTOVOICE STUDY TO EXPLORE THE PHYSICAL DISABILITY EXPERIENCE IN OLDER ADULTS WITH MCI/EARLY DEMENTIA

**DOI:** 10.1093/geroni/igac059.1494

**Published:** 2022-12-20

**Authors:** Emerald Jenkins, Janiece Taylor, Allyson Evelyn-Gustave, Erika Hornstein, Jennifer Wolff, George Rebok, Sarah Szanton

**Affiliations:** Johns Hopkins University, Baltimore, Maryland, United States; Johns Hopkins University, Baltimore, Maryland, United States; Johns Hopkins University, Baltimore, Maryland, United States; Johns Hopkins University, Baltimore, Maryland, United States; Johns Hopkins Bloomberg School of Public Health, Baltimore, Maryland, United States; Johns Hopkins Bloomberg School of Public Health, Baltimore, Maryland, United States; Johns Hopkins University School of Nursing, Baltimore, Maryland, United States

## Abstract

Adults aging with mild cognitive impairment (MCI)/early dementia often experience physical disability, yet most research is focused on cognitive or physical disability rather than both. Understanding the experiences of this population and their care partners will help identify unmet needs and potential solutions. The purpose of this study is to explore perceptions of daily experiences relevant to aging in place in this population. We used human-centered design, critical ethnography, photos, written narratives, and semi-structured interviews with community-dwelling care partners (mean age: 63 years (SD= 7.48)) and older adults with MCI/early dementia and physical disability (mean age: 84.5 years (SD =2.60)) (total n= 9). Example participant insights included: preferred nonpharmacological remedies for pain, environmental constraints resulting in further isolation, systems of reminders relevant to the older adults’ preferences, and the importance of shared dyadic activities. This study demonstrates participants’ lived experiences and challenges faced and informs tailoring of an evidenced-based intervention.

